# Editorial: Tumour microenvironment heterogeneity in hematological malignancies

**DOI:** 10.3389/fonc.2023.1295996

**Published:** 2023-10-09

**Authors:** Silvia Deaglio, Ingo Ringshausen

**Affiliations:** ^1^ Department of Medical Sciences, University of Turin, Turin, Italy; ^2^ Wellcome Trust/Medical Research Council (MRC) Cambridge Stem Cell Institute, Cambridge, United Kingdom; ^3^ Department of Haematology, Jeffrey Cheah Biomedical Centre, University of Cambridge, Cambridge, United Kingdom

**Keywords:** lymphoma, CLL (chronic lymphocytic leukemia), ALL - acute lymphoblastic leukemia, TME (tumor microenvironment), Hodgkin lymphoma, hairy cell leukemia (HCL)

Decades of cancer research have focused on identifying oncogenic drivers of transformation and understanding their effects on a molecular level. This ‘cancer cell centric’ approach has led to the development of numerous targeted therapies for the treatment of solid and blood cancers, which in general seem to be better tolerated with fewer systemic side effects than conventional chemotherapies. Unfortunately, the plasticity of cancer cells, enabeled by genomic instability and cell proliferation, remain common problems that affect such treatments and resistance to therapies continues to be a major obstacle in curing cancer. This has supported a more holistic view on cancer as a disease of tissues, rather than a pure cell intrinsic problem of gene-rearrangements and put the tumour microenvironment (TME) in the limelight of cancer research. In the past decade, our knowledge of the TME has grown exponentially, acknowledging that immune-, mesenchymal- and endothelial cells are not innocent bystander cells, but actively contribute to the transformation, progression, and therapy-resistance of cancer cells. A significant amount of TME work was then dedicated to solid cancers, and for a long time, research on blood cancers remained focused on the cell intrinsic mechanism of transformation. Therefore, for a long period of time, knowledge of the TME in epithelial cancers seemed more detailed than that of blood cancers. However, in recent years, further evidence has been generated, demonstrating that haematological malignancies have a similar capacity to actively reprogram cells of the TME. This knowledge has enabled the development of novel therapies, such as CAR-T cells and bispecific antibodies, which have started to transform the clinical management of patients with blood cancers.

This thematic Research Topic of *Frontiers in Oncology* is fully focused on discussing these topics in different haematological malignancies, ranging from B cell precursor acute lymphoblastic leukaemia (BCP-ALL) (Garcia-Gimenez and Richardson) to Hodgkin’s lymphoma (HL) (Ferrarini et al.), diffuse large B cell lymphoma (DLBCL) (Jayawant et al.), chronic lymphocytic leukaemia (CLL) (O’Donnell et al.,Martinez et al.), and hairy cell leukaemia (HL) (Gargiulo et al.).

Garcia-Gimenez and Richardson delineate microenvironmental changes in childhood B cell precursor acute lymphoblastic leukaemia (BCP-ALL) Garcia-Gimenez and Richardson. Peculiarly, BCP-ALL is believed to originate from pre-leukemic cells that require a fist transforming hit *in utero*. Strikingly, ETV6::RUNX1 translocations can be found in approximately 1-5% of healthy new-borns, while the frequency of the corresponding ALL is 100-fold less, indicating that ETV6::RUNX1 has low oncogenic potential and further genetic hits are required to fully establish the disease. Since foetal and postnatal blood development are organised in different compartments, this indicates that TME interactions are fundamentally different for pre-malignant cells and fully transformed ALL blasts. The authors delineate how pathogen-induced inflammation and epigenetic changes further drive transformation, which also impinges on TME interactions.

Ferrarini et al. discuss the role of macrophages in the context of Hodgkin lymphoma, and their implications for prognosis and therapy. The conclusions from this analytical review of the literature are that monocytes constitute a highly heterogeneous population that exerts different roles in the TME, mostly depending on their spatial localization and connection with the tumoral component. Importantly, following a simple parameter such as the lymphocyte-to-monocyte (LMR) ratio in the peripheral blood and the macrophage subtype composition in tissues has prognostic implications and can predict response to immunotherapy. It is therefore very important to study the circulating and residential myeloid population with specific markers to infer conclusions on their functional roles. Basic science studies are also unravelling multiple extracellular molecular circuits that involve macrophages, tumour cells and T cells. These studies may have functional implications and may direct therapeutic choices, as is the case for PD-1/PD-L1 targeting antibodies.

Jayawant et al. have applied an orthotopic approach to genomic studies to disentangle the contribution of genetic events and microenvironmental stimuli in Diffuse-large-B cell lymphoma (DLBCL). Focusing on a key transcription factor, namely the NF-κB, the authors developed a Flow cytometry-based method of NF-κB fingerprinting and identified significant differences in the expression of NF-κB-subunits, namely RelA, which did not align with established genomic classifiers. They then elegantly integrated published models of TLR and BCR signalling and recurrent mutations in DLCBCL in their work and demonstrated that this approach enables the accurate prediction of heterogeneous response to the TME, high-lightening that TME signals can have dominant effects over gene mutations.

The important role of NF-κB for the pathogenesis of Chronic Lymphocytic Leukaemia (CLL) is highlighted by O’Donnell et al. The signalling pathways driving proliferation and disease progression in this B cell lymphoma have extensively been studied and importantly, most of these pathways converge on NF-κB. Since upstream regulators originate from different sources in the TME, CLL has become a model disease for developing TME-targeted therapies. The authors of this paper review the current knowledge of canonical and non-canonical NF-κB signalling and how this knowledge has supported the development of novel therapies.

Martinez et al. proposes a research article that delves into the complex mechanisms of therapy resistance in CLL, a topic that is rapidly becoming the new CLL frontier. Emerging information led to the hypothesis that microenvironmental connections happening in the lymph node may tip the balance between pro- and anti-apoptotic molecules in favour of the latter, increasing the expression of anti-apoptotic molecules, such as Mcl1 and BCL-X_L_. Resistance occurs because these players are not targeted by the Bcl2 inhibitor venetoclax. In the lymph node microenvironment, CLL cells are in contact with different microenvironmental populations, which induce the expression of several molecules including the sphingosine kinases (SPHK). Since there are oral inhibitors of these enzymes in clinical trials, here, Gamberale and colleagues go a step further, by showing that co-culture of activated T lymphocytes with CLL cells not only induces Mcl1 and BCL-X_L_ expression but also SPHK and that the use of SPHK inhibitors prevents modulation of these molecules, opening the field to future therapeutic implications.

Gargiulo et al. explore the HCL microenvironment, which is typically located in the bone marrow and in the spleen, where leukemic cells are strongly attracted through the CXCL21/CXCR4 axis. In this comprehensive review, the authors examine old and novel therapeutic approaches used in the disease and discuss how they may affect relevant microenvironmental signals, ultimately resulting in leukemic cell apoptosis. They conclude that improvement in the treatment of this condition will only come by combining multiple approaches, as the microenvironment is made of many different cell types providing different survival signals to the HCL cell.

These six papers highlight the complexity of tumour-TME interactions in haematological malignancies and indicate how this knowledge can translate into patient care. It is expected that future work will focus on understanding the reciprocal interactions between tumour and non-tumour cells and enable hitherto unknown therapeutic approaches, further refining treatments for patients with haematological malignancies.

## Author contributions

IR: Writing – original draft. SD: Writing – original draft.

